# ALASKA NATIVE SUCCESSFUL AGING IN NORTHWEST ALASKA: AGING IN A GOOD WAY STARTS WITH YOUR FAMILY

**DOI:** 10.1093/geroni/igac059.1058

**Published:** 2022-12-20

**Authors:** Zayla Asquith-Heinz, Jordan Lewis, Steffi Kim

**Affiliations:** University of Minnesota Medical School, Minneapolis, Minnesota, United States; University of Minnesota Medical School, Duluth, Minnesota, United States; University of Minnesota, Minneapolis, Minnesota, United States

## Abstract

Alaska Natives (AN) view aging from a holistic perspective. One of the challenges of researching with cultural groups is the lack of data, or research, on culture and aging. This research explored successful aging from an AN perspective. A community-based participatory research (CBPR) model was used to engage participants at every stage of the research process. Semi-structured interviews were conducted with 16 AN men and 25 women. Kleinman’s Explanatory Model of Illness was adapted to gain a sense of the beliefs about aging and to guide the data analysis to establish an AN understanding of successful aging or attaining “Eldership” in Northwest Alaska. The foundation of the Norton Sound southern sub-region Model of Successful Aging is family, which contributed to Elders’ feelings of emotional well-being, ability to engage in their Native Way of life, maintain their physical health, and continue spiritual practices.

